# Outcomes of Patients Lost to Follow-up in African Antiretroviral Therapy Programs: Individual Patient Data Meta-analysis

**DOI:** 10.1093/cid/ciy347

**Published:** 2018-06-08

**Authors:** Frédérique Chammartin, Kathrin Zürcher, Olivia Keiser, Ralf Weigel, Kathryn Chu, Agnes N Kiragga, Cristina Ardura-Garcia, Nanina Anderegg, Christian Laurent, Morna Cornell, Hannock Tweya, Andreas D Haas, Brian D Rice, Elvin H Geng, Matthew P Fox, James R Hargreaves, Matthias Egger

**Affiliations:** 1 Institute of Social and Preventive Medicine, University of Bern; 2 Institute of Global Health, University of Geneva, Switzerland; 3 Faculty of Health, Witten/Herdecke University, Witten, Germany; 4 Lighthouse Trust, Lilongwe, Malawi; 5 Department of Surgery, University of Cape Town, South Africa; 6 Infectious Diseases Institute, College of Health Sciences, Makerere University, Kampala, Uganda; 7 Liverpool School of Tropical Medicine, United Kingdom; 8 Institut de Recherche pour le Développement, Inserm, Univ Montpellier, Recherches Translationnelles sur le VIH et les Maladies Infectieuses, Montpellier, France; 9 Centre for Infectious Disease Epidemiology and Research, School of Public Health and Family Medicine, University of Cape Town, South Africa; 10 Department of Social and Environmental Health Research, Faculty of Public Health and Policy, London School of Hygiene and Tropical Medicine, United Kingdom; 11 Division of Infectious Diseases, HIV and Global Medicine, Department of Medicine, University of California, San Francisco; 12 Health Economics and Epidemiology Research Office, Faculty of Health Sciences, University of the Witwatersrand, Johannesburg, South Africa; 13 Departments of Epidemiology and Global Health, Boston University School of Public Health, Massachusetts

**Keywords:** HIV, antiretroviral therapy, loss to follow-up, mortality, sub-Saharan Africa

## Abstract

**Background:**

Low retention on combination antiretroviral therapy (cART) has emerged as a threat to the Joint United Nations Programme on human immunodeficiency virus (HIV)/AIDS (UNAIDS) 90-90-90 targets. We examined outcomes of patients who started cART but were subsequently lost to follow-up (LTFU) in African treatment programs.

**Methods:**

This was a systematic review and individual patient data meta-analysis of studies that traced patients who were LTFU. Outcomes were analyzed using cumulative incidence functions and proportional hazards models for the competing risks of (*i*) death, (*ii*) alive but stopped cART, (*iii*) silent transfer to other clinics, and (*iv*) retention on cART.

**Results:**

Nine studies contributed data on 7377 patients who started cART and were subsequently LTFU in sub-Saharan Africa. The median CD4 count at the start of cART was 129 cells/μL. At 4 years after the last clinic visit, 21.8% (95% confidence interval [CI], 20.8%–22.7%) were known to have died, 22.6% (95% CI, 21.6%–23.6%) were alive but had stopped cART, 14.8% (95% CI, 14.0%–15.6%) had transferred to another clinic, 9.2% (95% CI, 8.5%–9.8%) were retained on cART, and 31.6% (95% CI, 30.6%–32.7%) could not been found. Mortality was associated with male sex, more advanced disease, and shorter cART duration; stopping cART with less advanced disease andlonger cART duration; and silent transfer with female sex and less advanced disease.

**Conclusions:**

Mortality in patients LTFU must be considered for unbiased assessments of program outcomes and UNAIDS targets in sub-Saharan Africa. Immediate start of cART and early tracing of patients LTFU should be priorities.

## Teaser

Retention on antiretrovirals is of concern in Africa. We analyzed outcomes in patients lost to follow-up: at 4 years after last contact, 22% had died, 23% had stopped therapy, 15% were in another clinic, and 32% could not be found.

Much progress has been made over the past 10 years with scaling up combination antiretroviral therapy (cART) in resource-limited settings: by July 2017, >20.9 million people were receiving cART [1]. Retention in care and adherence to cART are crucial for viral suppression and, therefore, for preventing human immunodeficiency virus (HIV)–related morbidity/mortality, HIV drug resistance, and onward transmission. Treatment programs’ effectiveness is therefore key for reaching the global 90-90-90 targets to end HIV/AIDS [2]. As documented repeatedly in systematic reviews of the literature, programs’ retention of HIV patients in resource-limited settings is a matter of concern; substantial proportions of patients are lost to follow-up (LTFU), particularly in the first year of cART [3–7].

Ignoring patients with LTFU undermines overall retention and underestimates program-level mortality as mortality is higher among patients LTFU [8–10]. In recent years, interest has grown in tracing patients LTFU to ascertain their vital and treatment status, and to bring patients back to care. We recently conducted a systematic review and meta-analysis of tracing studies of HIV-infected patients LTFU after starting cART in sub-Saharan Africa [11]. We found that mortality among patients LTFU had declined in more recent years, whereas undocumented (silent) transfer and treatment interruption increased. The previous study [11] was based on published aggregate data, which precluded analyses of the time to outcomes and the identification of the patient characteristics associated with different outcomes.

The present study extends the earlier work by analyzing individual patient data (IPD) from tracing studies identified in the systematic review. We estimated the cumulative incidence of death, stopping of cART, silent transfer to another care provider, and retention in care on cART, and examined factors associated with mortality and other tracing outcomes.

## METHODS

### Identification of Eligible Studies

The literature search and inclusion criteria are described in detail elsewhere [11]. In brief, we searched 3 databases (PubMed, Embase, and Latin American & Caribbean Health Sciences Literature) to identify eligible studies in sub-Saharan Africa published from 1 January 2009 to 31 December 2015. Studies where patients who started cART for their own health (adults or children fulfilling respective criteria of lifelong cART) were LTFU and then actively traced to establish their vital status were eligible. We excluded studies in tuberculosis patients and those focusing on prevention of mother-to-child transmission programs or postexposure prophylaxis. For the current study, we also excluded studies from South Africa that linked clinical databases with civil registry to identify deaths because only data on mortality but no other outcome were collected in these studies. The search of the bibliographic databases was complemented by reviewing the abstracts of the Conference on Retroviruses and Opportunistic Infections (2014–2015); the Conference on HIV Pathogenesis and Treatment of the International AIDS Society (2009–2015), and the International AIDS Conference (2010–2014).

### Request for Individual Patient Data

We contacted the authors of the 24 eligible studies [12–35] identified in the systematic review [11]. The data from abstracts were generally too sparse to determine eligibility, but abstract authors were also asked to contribute individual-level data, including patient characteristics (sex, date of birth, clinical stage at cART initiation, date of cART initiation, date of last contact), laboratory data (CD4 cell count at cART initiation), and tracing outcomes with their dates of occurrence and ascertainment. Eligibility was determined based on the data received. The data were cleaned in collaboration with the original investigators.

### Statistical Analysis

The studies considered a patient as LTFU if he or she did not return within 2 weeks to 3 months after the next scheduled appointment. Tracing outcomes were classified as “died,” “stopped cART” (ie, found alive but stopped cART), “transferred to other clinic,” “retained on cART” (found alive without having been transferred nor stopped cART), and “not found.” The group retained on cART includes patients erroneously classified as LTFU and intermittent attenders who came back to the clinic after a “treatment gap.” In the analysis, we considered all patients as being LFTU right after their last clinic visit and we defined time to event as the time between the last clinic visit and the date of the occurrence of the event (if available) or the date of its ascertainment (otherwise). We calculated nonparametric cumulative incidence functions to describe the probability of an outcome over time in the presence of the competing risks.

Subdistribution hazard models [36] were fitted to assess the association of patient characteristics with the different tracing outcomes. These models estimate the association of covariates on the cumulative incidence of an outcome, while accounting for competing events. We stratified our models to account for cohort heterogeneity by allowing cohort-specific baseline hazards [37]. The following patient characteristics were included as model covariates at the time of cART initiation: sex, World Health Organization (WHO) clinical stage (I–II, III, IV), and CD4 cell count (<50, 50–99, and ≥100 cells/µL). In addition, we included the calendar period (before 2009 and 2009 or later), age (<16, 16–29, 30–39, and ≥40 years) and the time on cART (<1, 1–2, and ≥2 years) at the last clinic visit. Missing WHO stage, CD4 cell counts, age, and time on cART were estimated through multiple imputations using a modified approach suitable for the subdistribution hazards model [38]. We imputed 20 datasets and pooled model parameter estimates using Rubin rules [39]. Sensitivity to imputation was assessed by comparing parameter estimates of models fitted on the imputed dataset with models fitted on the complete cases [40]. We assessed model fit using the Akaike information criterion and proportionality of hazards by inspecting model residuals against failure time and explored interactions between model covariates. We used a random intercept logistic regression model to examine the influence of the period between loss to follow-up and ascertainment of outcomes on the probability of remaining LTFU (outcome “not found”).

We compared the characteristics of published studies that provided IPD data with the studies that did not. Furthermore, we used random intercept logistic meta-regression models to compare mortality, transfer to another clinic, and stop cART estimates published in the respective articles between studies included and not included in the IPD meta-analysis.

For cohorts that provided data on both patients retained in care and patients LTFU, we calculated nonparametric cumulative incidence functions for the 2 groups separately to compare their mortality after the start of cART (again accounting for the presence of competing risks). Finally, we repeated analyses excluding a large study from Lilongwe, Malawi [25].

All analyses were carried out using R statistical software (version 3.3.2) [41], including the packages “mstate” for cumulative incidence analysis, “crrSC” for stratified subdistribution hazard modeling, and “smcfcs” for multiple imputation. Results are shown as cumulative probabilities or subdistribution hazard ratios with 95% confidence intervals (CIs).

## RESULTS

**Figure 1.**
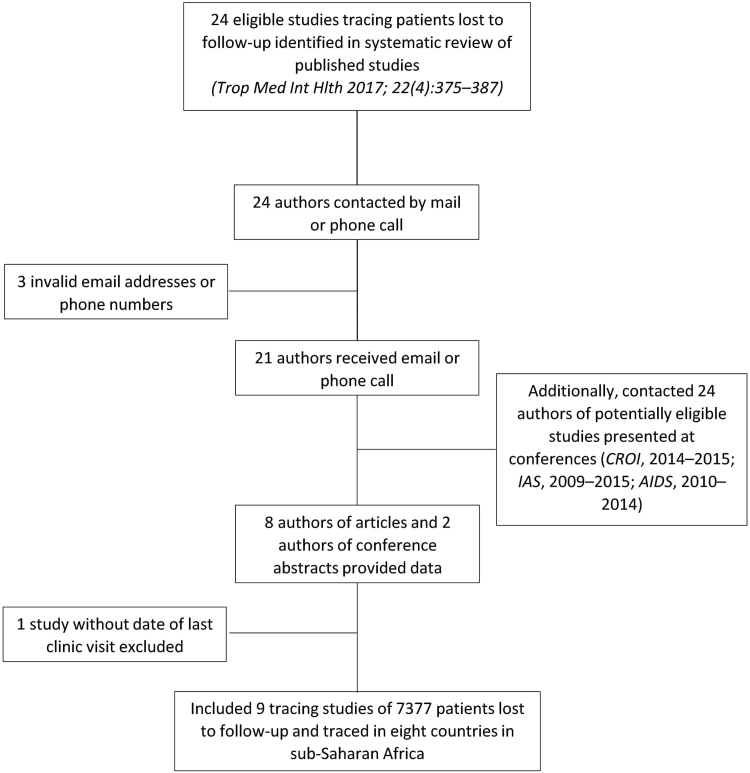
Flow of identifying eligible studies that contributed individual patient data.

The authors of 22 of the 24 eligible articles responded by electronic mail or phone. In addition, 24 authors of potentially eligible abstracts were contacted (Figure 1). Seven authors of published articles [12–14, 21–25] and 2 abstract [42–43] authors provided data on 7377 patients who started cART in a treatment program in 1 of 8 countries in East Africa (Kenya, Tanzania, Uganda), Southern Africa (Malawi, Mozambique, Zambia, Zimbabwe), or Central Africa (Cameroon) and were later LTFU and traced. All sites were involved in the routine treatment and care of HIV-infected patients.

Sociodemographic and clinical features of patients, together with cohort characteristics, are summarized in Table 1. One study included adults and children [13], 1 study included children only [24], and 6 studies included adults aged ≥16 years only. Overall, patients’ median age at last clinic visit was 34 years (interquartile range [IQR], 28–41 years) and 4148 of 7377 patients (56.2%) were female. The median CD4 cell count at cART initiation was 129 cells/µL (IQR, 54–216 cells/µL); most patients were in WHO clinical stage III or IV (5090 [69.0%]). CD4 cell counts were missing for 2987 patients (40.5%) and 1448 patients (19.6%) had a missing WHO clinical stage. The majority of patients were lost in the first 6 months after cART initiation: the median time on cART was 162 days overall; it ranged from 35 days in the study from Zimbabwe [42] to 328 days in the study from Kenya [13] (Table 1).

**Table 1.** Characteristics of the Tracing Studies Contributing Data to Individual Patient Data Meta-analysis

Characteristic	East Africa			Southern Africa					Central Africa	All Studies
	Rachlis, 2015 [13]	Kiragga, 2013 [14]	Geng, 2015 [12]	Tweya, 2013 [25]	Ardura-Garcia, 2015 [24]	Caluwaerts, 2009 [27]	Gunguwo, 2012 [42]	Kato, 2013 [43]	Mben, 2012 [21]	
Country	Kenya	Uganda	Kenya, Uganda, Tanzania	Malawi	Malawi	Mozambique	Zimbabwe	Zambia	Cameroon	
No. of patients	881	163	579	4558	201	594	110	53	238	7377
No. of women	563 (63.9)	90 (55.2)	373 (64.4)	2440 (53.5)	104 (51.7)	331 (55.7)	71 (64.5)	25 (47.2)	151 (63.4)	4148 (56.2)
Median age, y^b^ (IQR)	35 (28–42)	37 (33–43)	34 (28–41)	35 (29–42)	9 (5–13)	32 (27–40)	37 (30–48)	35 (30–42)	37 (30–42)	34 (28–41)
No. with missing age	2 (0.2)	0 (0)	11 (1.9)	0 (0)	0 (0)	0 (0)	0 (0)	0 (0)	0 (0)	13 (0.2)
Median CD4 count^a^, cells/µL (IQR)	137 (58–234)	87 (26–195)	150 (62–234)	127 (54–210)	262 (135–512)	105 (46–176)	106 (40–234)	144 (88–230)	118 (46–199)	129 (54–216)
No. with missing CD4 count	300 (34.1)	43 (26.4)	104 (18)	2160 (47.4)	53 (26.4)	279 (47)	48 (43.6)	0 (0)	0 (0)	2987 (40.5)
WHO clinical stage^a^										
I–II	355 (40.3)	34 (20.9)	239 (41.3)	0 (0)	13 (6.5)	149 (25.1)	23 (20.9)	26 (49.1)	0 (0)	839 (11.4)
III	315 (35.8)	61 (37.4)	219 (37.8)	2597 (57)	138 (68.7)	264 (44.4)	36 (32.7)	25 (47.2)	0 (0)	3655 (49.5)
IV	117 (13.3)	68 (41.7)	78 (13.5)	968 (21.2)	41 (20.4)	110 (18.5)	51 (46.4)	2 (3.8)	0 (0)	1435 (19.5)
Missing	94 (10.7)	0 (0)	43 (7.4)	993 (21.8)	9 (4.5)	71 (12)	0 (0)	0 (0)	238 (100)	1448 (19.6)
Median No. of days on cART^b^ (IQR)	328 (98–942)	146 (46–327)	59 (14–245)	166 (40–457)	272 (77–681)	109 (14–365)	35 (14–84)	179 (43–351)	122 (28–357)	162 (35–454)
Median No. of days from LTFU to tracing (IQR)	1332 (838–1812)	1503 (803–1681)	612 (320–960)	782 (170–1567)	579 (218–1474)	1461 (523–1592)	321 (85–1461)	774 (745–830)	251 (85–524)	858 (242–1550)
Definition of LTFU (time since last scheduled visit)	3 mo	3 mo	3 mo	3 wk	3 wk	2 mo	3 mo	Not reported	1 mo	
Tracing method	Home visits	Phone calls & home visits	Phone calls & home visits	Phone calls & home visits	Phone calls & home visits	Home visits	Phone calls & home visits	Phone calls & home visits	Phone calls & home visits	

Data are presented as No. (%) unless otherwise indicated.

Abbreviations: cART, combination antiretroviral therapy; IQR, interquartile range; LTFU, loss to follow-up; WHO, World Health Organization.

^a^At cART initiation.

^b^At last clinic visit.

All programs traced patients by home visits, or by phone calls and home visits. The median number of days from LTFU to tracing ranged from 82 days in the study from Cameroon [21] to 588 days in Kenya [13], and was 327 days overall (Table 2). A total of 1606 (21.8%) deaths were identified; 1667 (22.6%) individuals were found to be alive but had stopped cART; 1094 (14.8%) had transferred to another clinic, and 2119 (28.7%) could not be found (Table 2). For outcome death, the date of death was available in most cases (in 1516 of 1608 deaths [94.3%]). For outcomes stopping cART and transfer to another clinic, the exact date the outcome occurred was generally unavailable and the date of ascertainment was used instead in 1673 of 1683 (99.4%) for stopping cART and in 966 of 1098 (88.0%) for silent transfer. In logistic regression, a longer delay between loss to follow-up and tracing was associated with an increase in the probability of the patient remaining LTFU (outcome “not found”). The odds ratio (OR) per standard deviation increase in the number of days between LTFU and tracing was 2.05 (95% CI, 1.95–2.15). The probability of the other outcomes was reduced accordingly.

**Table 2.** Outcomes Ascertained in the Tracing Studies Contributing Data to Individual Patient Data Meta-analysis

Outcome	East Africa			Southern Africa					Central Africa	All Studies
	Rachlis, 2015 [13]	Kiragga, 2013 [14]	Geng, 2015 [12]	Tweya, 2013 [25]	Ardura-Garcia, 2015 [24]	Caluwaerts, 2009 [27]	Gunguwo, 2012 [42]	Kato, 2013 [43]	Mben, 2012 [21]	
Country	Kenya	Uganda	Kenya, Uganda, Tanzania	Malawi	Malawi	Mozambique	Zimbabwe	Zambia	Cameroon	
No. of patients	881	163	579	4558	201	594	110	53	238	7377
Median No. of days from LTFU to tracing (IQR)	588 (414–833)	572 (339–808)	404 (92–676)	206 (29–781)	85 (56–157)	396 (2–672)	234 (24–343)	481 (172–622)	82 (25–150)	327 (40–746)
No. of deaths	160 (18.2)	35 (21.5)	157 (27.1)	952 (20.9)	17 (8.5)	117 (19.7)	55 (50.0)	15 (28.3)	98 (41.2)	1606 (21.8)
No. stopping cART	457 (51.9)	0 (0)	0 (0)	984 (21.6)	41 (20.4)	46 (7.7)	5 (4.5)	15 (28.3)	119 (50.0)	1667 (22.6)
No. transferred	17 (1.9)	24 (14.7)	188 (32.5)	740 (16.2)	41 (20.4)	49 (8.2)	4 (3.6)	10 (18.9)	21 (8.8)	1094 (14.8)
No. retained on cART	10 (1.1)	0 (0)	152 (26.3)	627 (13.8)	75 (37.3)	3 (0.5)	16 (14.5)	8 (15.1)	0 (0)	891 (12.1)
No. lost to follow-up	237 (26.9)	104 (63.8)	82 (14.2)	1255 (27.5)	27 (13.4)	379 (63.8)	30 (27.3)	5 (9.4)	0 (0)	2119 (28.7)

Data are presented as No. (%) unless otherwise indicated.

Abbreviations: cART, combination antiretroviral therapy; IQR, interquartile range; LTFU, loss to follow-up.

**Figure 2.**
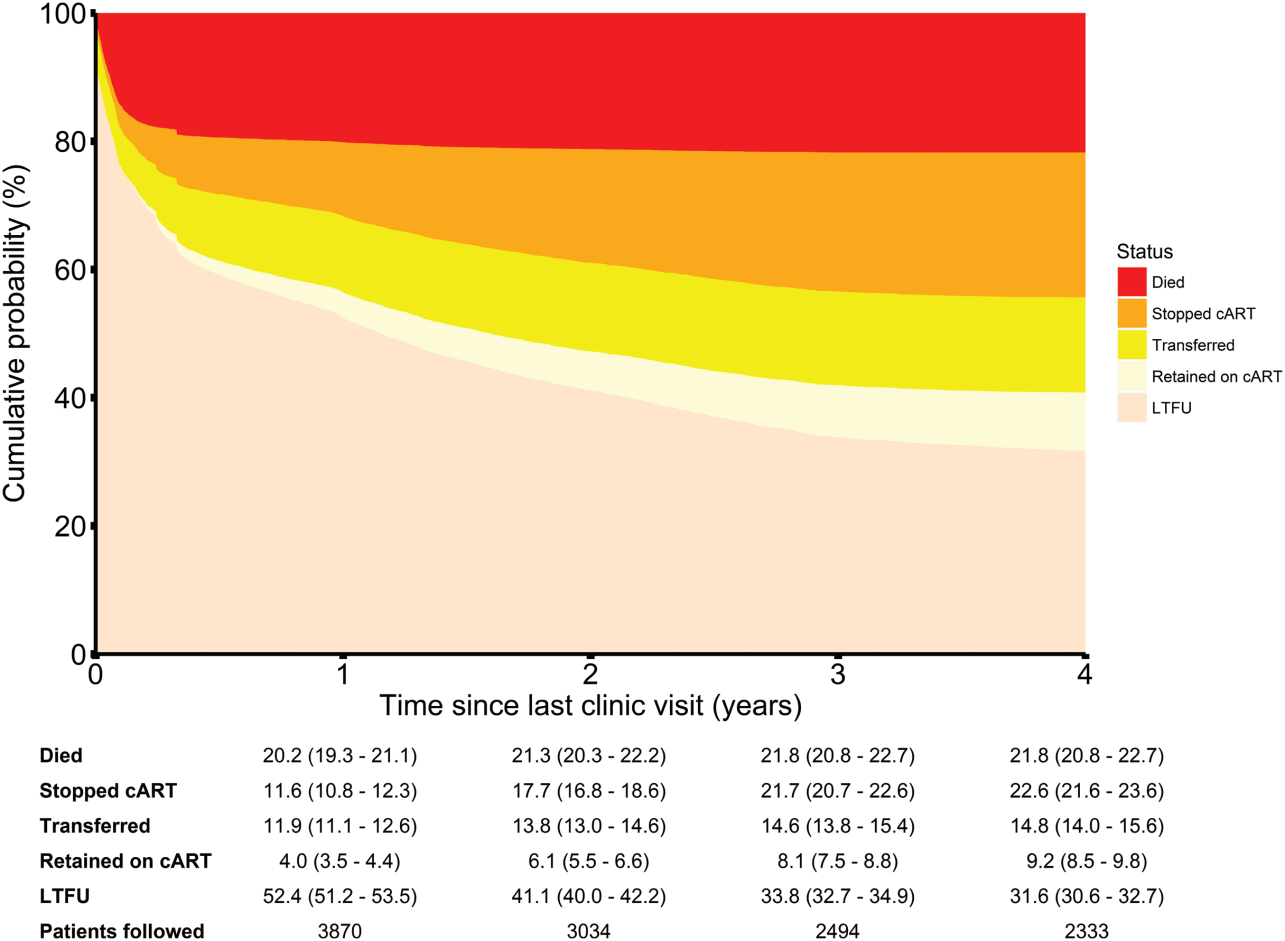
Cumulative incidence plot of the outcomes in patients lost to follow-up (LTFU), including death, stop of combination antiretroviral therapy (cART), transfer to another clinic, and retention on cART. Cumulative probabilities, together with 95% confidence intervals, are given for each tracing outcome at 1–4 years since the last clinic visit.

Figure 2 shows the cumulative incidence functions for each tracing outcome stacked on top of each other, together with a table showing point estimates and 95% CIs at 1, 2, 3, and 4 years after the last clinic visit. Four years after the last clinic visit, an estimated 21.8% (95% CI, 20.8%–22.7%) had died, 22.6% (21.6%–23.6%) were alive but had stopped cART, 14.8% (14.0%–15.6%) had transferred to another clinic, 9.2% (8.5%–9.8%) were considered as retained on cART, and 31.6% (30.6%–32.7%) could not be found.

The results of the subdistribution-specific hazard models for the different tracing outcomes are shown in Table 3. The inclusion of an interaction term between gender and clinical stage further improved the fit of the models for the mortality outcome. Mortality was associated with male sex, lower CD4 count, and more advanced clinical stage at cART initiation. Mortality was also associated with shorter duration on cART at the time of the last clinic visit, older age, and a last clinic visit before 2009. Stopping cART was associated with higher CD4 count, less advanced clinical stage, and longer duration on cART. Silent transfer to another clinic was associated with female sex, less advanced clinical stage, and a more recent last visit (2009 or later). Finding patients alive and on cART in the program was associated with higher CD4 counts, less advanced clinical stage, last visit before 2009, longer time on cART, and adult age. The results from the complete case analysis, without imputation of missing CD4 cell counts, WHO clinical stage, age, and time on cART at LTFU were similar (Supplementary Table 1). When excluding the large study from Lilongwe, Malawi [25], results were similar to the main analysis (Supplementary Tables 2 and 3).

**Table 3.** Subdistribution Hazard Ratios for Tracing Outcomes Death, Stop of Combination Antiretroviral Therapy (cART), Silent Transfer, and Retention on cART

Characteristic	Death	Stop of cART	Silent Transfer	Retained on cART
	SHR (95% CI)	SHR (95% CI)	SHR (95% CI)	SHR (95% CI)
Sex				
Male	1	1	1	1
Female	0.62 (.44–.88)	0.97 (.87–1.07)	1.32 (1.17–1.50)	1.03 (.90–1.18)
CD4 count^a^ (cells/µL)				
<50	1	1	1	1
50–99	0.75 (.64–.88)	1.52 (1.21–1.90)	1.13 (.86–1.49)	0.88 (.66–1.19)
≥100	0.40 (.35–.45)	1.77 (1.47–2.13)	1.17 (.93–1.47)	1.47 (1.17–1.84)
WHO clinical stage^a^				
I–II	1	1	1	1
III	1.23 (.93–1.63)	0.77 (.64–.91)	0.88 (.71–1.09)	0.77 (.60–1.00)
IV	1.57 (1.17–2.10)	0.64 (.51–.80)	0.71 (.56–.91)	0.71 (.53–.95)
Last clinic visit				
Before 2009	1	1	1	1
2009 or later	0.76 (.66–.87)	1.04 (.92–1.18)	1.56 (1.36–1.80)	0.33 (.27–.40)
Age^b^ (y)				
<16	1	1	1	1
16–29	1.73 (.92–3.25)	0.99 (.70–1.39)	0.71 (.35–1.45)	1.68 (1.00–2.82)
30–39	2.21 (1.18–4.15)	0.89 (.63–1.25)	0.84 (.41–1.72)	1.79 (1.04–3.08)
≥40	2.84 (1.52–5.32)	0.75 (.53–1.06)	0.91 (.45–1.87)	1.79 (1.04–3.09)
Time on cART^b^ (y)				
<1	1	1	1	1
1–2	0.51 (.44–.60)	1.28 (1.13–1.45)	1.02 (.87–1.20)	1.89 (1.62–2.21)
≥2	0.44 (.36–.53)	1.14 (1.00–1.30)	1.05 (.87–1.28)	1.94 (1.60–2.37)
Interaction^a^				
Female clinical stage I–II	1			
Female clinical stage III	1.32 (.91–1.92)			
Female clinical stage IV	1.73 (1.17–2.56)			

Abbreviations: cART, combination antiretroviral therapy; CI, confidence interval; SHR, subdistribution hazard ratio; WHO, World Health Organization.

^a^At cART initiation.

^b^At last clinic visit. All models are stratified by cohort. Parameter estimates are pooled estimates from models fitted to 20 imputed datasets. Each model is fitted for the tracing outcome of interest, accounting for the alternative outcomes as competing risks.

The 7 published studies that provided IPD were broadly representative of all published studies: the 3 regions East Africa, Southern Africa, and Central Africa were represented, and included and excluded studies were similar in their sex and age distributions and study periods. Included studies all conducted home visits for tracing patients and were done in urban settings, whereas some excluded studies used telephone tracing only, and a few excluded studies were from rural settings (Supplementary Table 4). Of note, the 7 studies contributed 7377 patients, which corresponds to 55.9% of the 13200 patients included in the 24 published studies. The comparison of the published aggregate data showed that there was little evidence for a difference in mortality, transfer to another clinic, and stop of cART between the included studies compared with the excluded ones (OR, 0.62 [95% CI, .29–1.33]; OR, 1.04 [95% CI, .29–1.33]; and OR, 1.59 [95% CI, .80–3.19], respectively).

**Figure 3.**
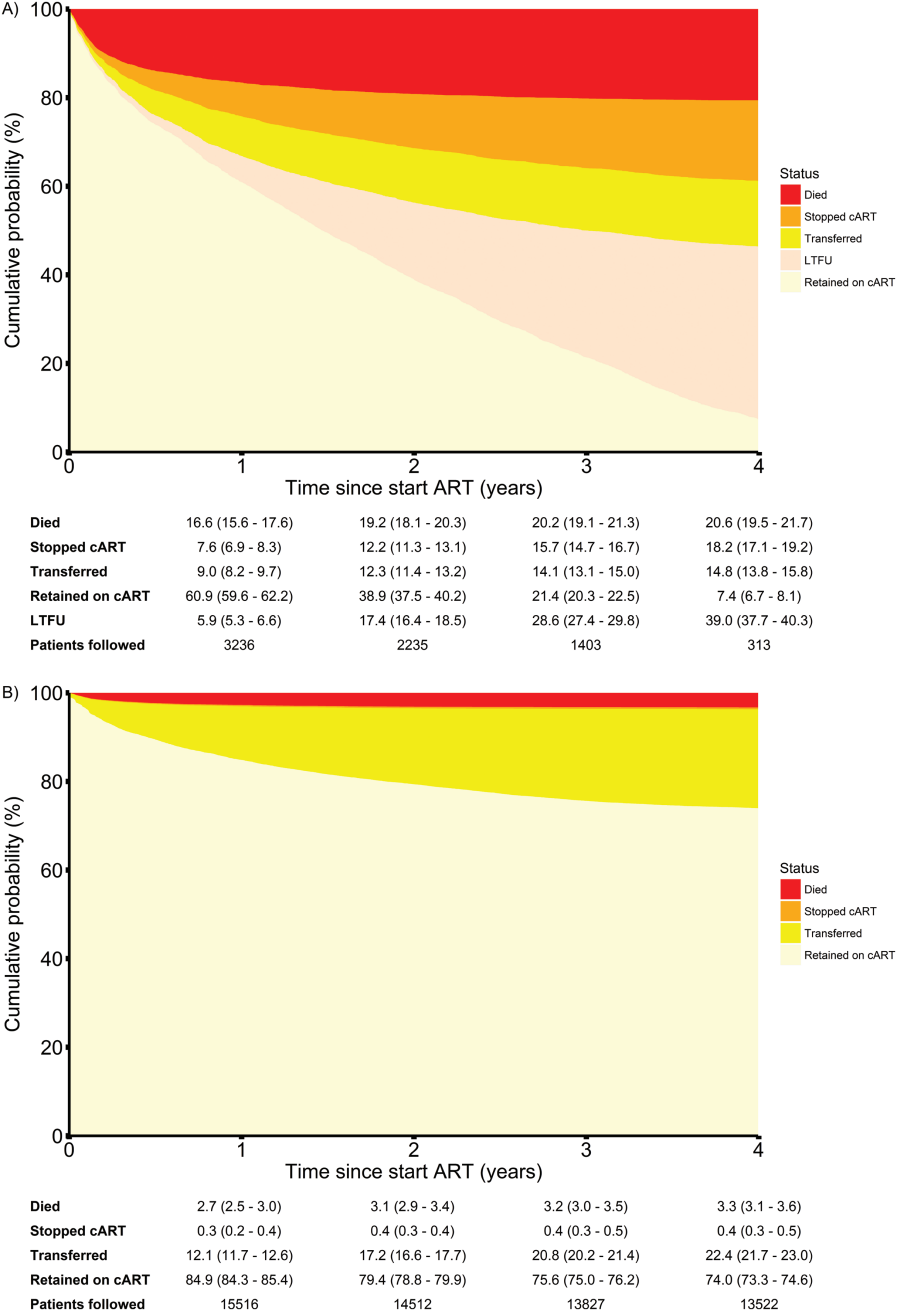
Cumulative incidence plot of outcomes for 5152 patients lost to follow-up (LTFU) after starting combination antiretroviral therapy (cART) (*A*) and 18285 patients retained in care (*B*) in 2 treatment programs in Malawi and Mozambique. Cumulative probabilities, together with 95% confidence intervals, are given for each outcome at 1–4 years after cART initiation.

Two cohorts [25, 27] from Malawi and Mozambique provided data on both patients retained in care (n = 18285) and patients LTFU (n = 5152). For those cohorts, the cumulative probability of death 4 years after the start of cART was estimated to be >6 times higher among LTFU patients compared to patients retained in care: 20.6% (95% CI, 19.5%–21.7%) vs 3.3% (95% CI, 3.1%–3.6%) (Figure 3).

## DISCUSSION

This collaborative study pooled individual-level data of almost 7500 patients from 9 antiretroviral treatment programs in 8 African countries to estimate mortality and other outcomes in patients LTFU. The results show that 4 years after the last clinic visit, about one-fifth of patients LTFU had died, a similar proportion had stopped treatment, one-sixth had silently transferred to another clinic, and about one-third were not found. As expected, patients were more likely to remain LTFU as the delay between LFTU and tracing increased. Mortality was associated with male sex and more advanced disease, silent transfer with female sex and less advanced disease, and stopping therapy with less advanced disease. Death, stopping cART, and unsuccessful tracing were all associated with shorter duration of cART at the time of LTFU.

The analysis of IPD is an important strength of our study, which made it possible to estimate the association of patient characteristics on clinical outcomes, including the CD4 cell count and WHO clinical stage at cART initiation, and the duration on cART before patients became LTFU. Such IPD meta-analyses have been described as the “yardstick” against which the quality of other reviews should be judged [44, 45]. Advantages of IPD meta-analyses include the possibility of conducting time to event analyses, harmonizing definitions and time points, but also the prevention of ecological bias where associations at the aggregate level do not reflect those at the individual level [46, 47]. Furthermore, the active involvement of investigators contributing data may enhance data quality, facilitate the provision of more recent data, and improve interpretation of results. Indeed, several datasets provided for this analysis were updated to include more patients, and the authors of the 9 studies [12–14, 21–25, 27, 42, 43] all contributed to this project. Another strength of this study was the comprehensive literature search, which covered several bibliographic databases and recent major conferences [11].

IPD meta-analyses also have disadvantages. IPD are obtained typically only from a proportion of all eligible studies. Selection bias is therefore possible if the studies contributing data are not representative of all studies. We addressed this issue by comparing the 7 published articles that were included in this analysis with the 15 that were excluded. We found that the included studies contributed more than half of all patients included in the published reports. Also, the characteristics of patients from included and excluded studies were similar, although CD4 cell count and clinical stage were not reported consistently in the published studies [11]. Finally, mortality and other tracing outcomes were similar among included and excluded studies.

Our analysis took the competing risks of death and other tracing outcomes into account. For example, patients who died could no longer transfer to another treatment program, and patients who transferred could not be recorded as a death in the clinic where cART was initiated. In standard Kaplan-Meier analyses, the follow-up time of those developing a competing event is simply censored, assuming that the probability of the outcome of interest is the same as that of comparable patients remaining under observation [48]. This assumption is invalid because the outcome of interest can no longer occur in those with the competing event. An analysis of mortality in HIV-infected patients on cART followed up in Zambia showed that the competing risk of death can substantially bias standard Kaplan-Meier life-table analyses of LFTU [49].

Our competing risk modeling approach accounted for between-study heterogeneity, including heterogeneity in study settings and size, by allowing for cohort-specific baseline hazards. Of note, between-study heterogeneity in baseline hazards also did not materially influence estimates of the cumulative incidence of the different outcomes. In particular, results were similar when excluding the large study by Tweya et al [25], which contributed 61.8% of all patients. This study, despite operating in a relatively well-resourced urban setting, can be assimilated to a real-world healthcare setting with routine data collection.

Silent transfers have increased with the scaling up of cART, likely due to the expansion of cART access and the availability of clinics nearer to patients’ home [11, 50]. We found that about 15% of patients who were LTFU in the clinic where they started cART transferred to another facility within 4 years. Retention in care is therefore underestimated in analyses of individual clinics or programs [51]. Clearly, improving communication between the clinics and programs, for example, through a national cART database and unique patient identifiers [52], is urgently needed for an accurate assessment of overall retention in care. On the other hand, we confirm the much higher risk of death among patients LTFU compared to those retained in care, and the fact that estimates of mortality that are based on patients in care and under observation through a single facility data system will underestimate mortality at the level of the program, that is, among all patients who started therapy [8, 9, 52].

The ratio of mortality between patients lost and not LTFU observed in our study for 2 treatment programs in Malawi [25] and Mozambique [27] can be directly used to correct mortality estimates for LTFU, based on the fact that mortality of all patients starting cART in a treatment program is a weighted average of mortality among patients lost and patients remaining in care [8]. In general, the risk factors for mortality identified in our study will be useful to refine methods to correct mortality for LTFU, for example by taking the lower risk of death in women, the association with the CD4 cell count and clinical stage at the start of cART, or the duration of cART at LTFU into account. Indeed, within the framework of the HIV Measurement & Surveillance of HIV Epidemics (MeSH) Consortium, we will be working on improving existing methods [14] and developing new applications, based on the findings of the present study.

The risk factors for death identified in this study are directly relevant to HIV care and treatment programs in sub-Saharan Africa. For example, time on cART at the time of LTFU was an important determinant of the mortality risk among patients LTFU. We showed that patients who initiated cART <1 year prior to being lost were at higher mortality risk. These patients, with low CD4 counts or advanced clinical stage, should therefore be prioritized for tracing, with stricter definitions for LTFU to trigger early action, so that they can be supported to remain in care and on cART as soon as possible. Furthermore, tracing should start soon after LTFU to reduce mortality, and the number of patients who are not found.

To conclude, our study showed that mortality and undocumented transfer were substantial among LTFU patients in sub-Saharan Africa. The results are useful to predict clinical outcomes in patients LTFU, and to assess program effectiveness, with less biased estimates of retention and mortality. We acknowledge that our results may not be generalizable to many settings in sub-Saharan Africa, and we recommend that cART programs trace patients LTFU and use results to estimate program-level outcomes. Indeed, to further improve our understanding of LTFU and the outcomes of patients LTFU, we are planning tracing studies using standardized methods and data collection within the framework of the International epidemiology Databases to Evaluate AIDS (IeDEA) [53], including studies in South Africa where mortality of patients not found by tracing can be ascertained trough linkage with the civil registry [52].

## Supplementary Data

Supplementary materials are available at *Clinical Infectious Diseases* online. Consisting of data provided by the authors to benefit the reader, the posted materials are not copyedited and are the sole responsibility of the authors, so questions or comments should be addressed to the corresponding author.

Supplemental Materials

## Notes

***Financial support.*** This study was funded by the Bill & Melinda Gates Foundation through the Measurement and Surveillance of HIV Epidemics Consortium and the National Institutes of Health (award number U01AI069924). O. K. was supported by professorship grant number 163878 and M. E. was supported by special project funding (award number 174281) from the Swiss National Science Foundation.

***Disclaimer.*** The funding sources had no involvement in the design of the study; the collection, analysis, and interpretation of data; the writing of the report; or the decision to submit the article for publication.

***Potential conflicts of interest.*** All authors: No potential conflicts of interest. All authors have submitted the ICMJE Form for Disclosure of Potential Conflicts of Interest. Conflicts that the editors consider relevant to the content of the manuscript have been disclosed.
