# Supplementary material for: Outcomes of Patients Lost to Follow-up in African Antiretroviral Therapy Programs: Individual Patient Data Meta-analysis
Source: Clin Infect Dis. 2018 Jun 8;67(11):1643–52. doi: 10.1093/cid/ciy347 (PMC6233676; doi:10.1093/cid/ciy347)
Supplement: Supplemental Materials [file ciy347_suppl_supplemental-materials.docx]

**Supplemental materials**

**Table S1**. Sub-distribution hazard ratios (SHR) for tracing outcomes death, stop of combination
antiretroviral therapy (cART), silent transfer, and retention on cART. Analysis based on 3508 patients
with complete CD4-cell count, clinical stage, age and time on cART.

|  | Death | Stop of cART | Silent Transfer | Retained on cART |
| --- | --- | --- | --- | --- |
|  | SHR (95% CI) | SHR (95% CI) | SHR (95% CI) | SHR (95% CI) |
| Sex |  |  |  |  |
| Male | 1 | 1 | 1 | 1 |
| Female | 0.48 (0.31-0.74) | 0.99 (0.86-1.15) | 1.36 (1.13-1.63) | 1.01 (0.83-1.22) |
| CD4* (cells/ µl) |  |  |  |  |
| < 50 | 1 | 1 | 1 | 1 |
| 50-99 | 0.79 (0.66-0.94) | 1.46 (1.14-1.87) | 1.16 (0.87-1.55) | 0.89 (0.62-1.27) |
| ≥ 100 | 0.41 (0.35-0.48) | 1.82 (1.50-2.21) | 1.14 (0.91-1.42) | 1.57 (1.22-2.01) |
| WHO clinical stage* |  |  |  |  |
| I-II | 1 | 1 | 1 | 1 |
| III | 1.17 (0.83-1.64) | 0.72 (0.58-0.90) | 0.75 (0.58-0.98) | 0.64 (0.47-0.89) |
| IV | 1.30 (0.91-1.87) | 0.62 (0.48-0.80) | 0.56 (0.41-0.77) | 0.62 (0.43-0.90) |
| Last clinic visit |  |  |  |  |
| < 2009 | 1 | 1 | 1 | 1 |
| ≥ 2009 | 0.75 (0.61-0.92) | 1.02 (0.84-1.23) | 1.62 (1.28-2.05) | 0.36 (0.27-0.49) |
| Age** (year) |  |  |  |  |
| < 16 | 1 | 1 | 1 | 1 |
| 16-29 | 1.39 (0.31-6.34) | 1.78 (0.82-3.87) | 0.57 (0.17-1.97) | 1.65 (0.90-3.02) |
| 30-39 | 1.67 (0.37-7.62) | 1.62 (0.74-3.57) | 0.60 (0.17-2.08) | 1.97 (1.03-3.78) |
| ≥ 40 | 2.16 (0.47-9.86) | 1.51 (0.68-3.33) | 0.66 (0.19-2.30) | 1.78 (0.92-3.46) |
| Time on cART** (year) |  |  |  |  |
| < 1 | 1 | 1 | 1 | 1 |
| 1-2 | 0.51 (0.42-0.64) | 1.39 (1.16-1.67) | 0.87 (0.68-1.11) | 1.58 (1.25-1.99) |
| ≥ 2 | 0.48 (0.37-0.64) | 1.26 (1.04-1.54) | 1.07 (0.77-1.48) | 1.63 (1.17-2.26) |
| Interaction |  |  |  |  |
| Female*clinical stage I-II | 1 |  |  |  |
| Female*Clinical stage III | 1.79 (1.13-2.84) |  |  |  |
| Female*Clinical stage IV | 2.76 (1.69-4.50) |  |  |  |

* at cART initiation, ** at last clinic visit

Supplementary Table S2: Cumulative probabilities for each tracing outcome at one to four years after cART initiation. Fitted on a subset of the data, excluding the study from Tweya et al [1].

|  | **Year 1** | **Year 2** | **Year 3** | **Year 4** |
| --- | --- | --- | --- | --- |
| Died | 19.1 ( 17.6 - 20.5 ) | 21.9 ( 20.3 - 23.4 ) | 23.2 ( 21.6 - 24.7 ) | 23.2 ( 21.6 - 24.8 ) |
| Stopped cART | 8.3 ( 7.2 - 9.3 ) | 18.9 ( 17.5 - 20.4 ) | 24.1 ( 22.5 - 25.6 ) | 24.2 ( 22.6 - 25.8 ) |
| Transferred | 7.1 ( 6.1 – 8.0 ) | 10.9 ( 9.7 – 12.0 ) | 12.5 ( 11.3 - 13.7 ) | 12.6 ( 11.3 - 13.8 ) |
| Retained on cART | 4.3 ( 3.5 – 5.0 ) | 7.1 ( 6.1 – 8.0 ) | 8.3 ( 7.2 - 9.3 ) | 9.4 ( 8.3 - 10.4 ) |
| LTFU | 61.3 ( 59.5 - 63.1 ) | 41.3 ( 39.5 - 43.1 ) | 32 ( 30.3 - 33.7 ) | 30.6 ( 28.9 - 32.4 ) |
| Patients followed | 1734 | 1166 | 903 | 893 |

Supplementary Table S3: Sub-distribution hazard ratios (SHR) for tracing outcomes death, stop of combination antiretroviral therapy (cART), silent transfer, and retention on cART. Model fitted on a subset of the data, excluding the study of Tweya at al [1].

|  | **Death** | **Stop of cART** | **Silent transfer** | **Retained on cART** |
| --- | --- | --- | --- | --- |
|  | SHR (95% CI) | SHR (95% CI) | SHR (95% CI) | SHR (95% CI) |
| Sex |  |  |  |  |
| Male | 1 | 1 | 1 | 1 |
| Female | 0.65 (0.46-0.91) | 0.92 (0.78-1.08) | 1.32 (1.05-1.66) | 0.96 (0.73-1.26) |
| CD4* (cells/ µl) |  |  |  |  |
| < 50 | 1 | 1 | 1 | 1 |
| 50-99 | 0.72 (0.57-0.9) | 1.46 (1.07-2) | 1.5 (0.97-2.31) | 0.56 (0.29-1.09) |
| ≥ 100 | 0.41 (0.34-0.51) | 1.83 (1.42-2.35) | 1.72 (1.21-2.45) | 1.9 (1.24-2.93) |
| WHO clinical stage* |  |  |  |  |
| I-II | 1 | 1 | 1 | 1 |
| III | 1.17 (0.84-1.63) | 0.8 (0.65-0.98) | 0.82 (0.63-1.06) | 0.8 (0.58-1.1) |
| IV | 1.46 (1.04-2.06) | 0.66 (0.5-0.88) | 0.61 (0.43-0.88) | 0.56 (0.35-0.91) |
| Last clinic visit |  |  |  |  |
| < 2009 | 1 | 1 | 1 | 1 |
| ≥ 2009 | 1.00 (0.54-1.83) | 2.45 (1.56-3.86) | 1.11 (0.66-1.86) | 0.33 (0.2-0.53) |
| Age** (year) |  |  |  |  |
| < 16 | 1 | 1 | 1 | 1 |
| 16-29 | 1.65 (0.89-3.05) | 0.97 (0.67-1.39) | 0.97 (0.48-1.94) | 2.19 (1.16-4.12) |
| 30-39 | 1.86 (1.01-3.42) | 0.99 (0.7-1.42) | 1.02 (0.5-2.09) | 1.93 (0.95-3.9) |
| ≥ 40 | 2.75 (1.5-5.05) | 0.75 (0.52-1.08) | 1.06 (0.51-2.19) | 1.17 (0.55-2.49) |
| Time on cART** (year) |  |  |  |  |
| < 1 | 1 | 1 | 1 | 1 |
| 1-2 | 0.64 (0.5-0.82) | 1.8 (1.47-2.19) | 1.36 (1.04-1.78) | 1.46 (1.04-2.06) |
| ≥ 2 | 0.54 (0.4-0.73) | 1.5 (1.24-1.82) | 1.55 (0.98-2.45) | 0.96 (0.59-1.57) |
| Interaction |  |  |  |  |
| Female*clinical stage I-II | 1 |  |  |  |
| Female*clinical stage III | 1.53 (1-2.34) |  |  |  |
| Female*clinical stage IV | 1.71 (1.09-2.68) |  |  |  |

* at cART initiation, ** at last clinic visit

Supplementary Table S4. Comparison of tracing studies included and excluded from the individual patient data (IPD) meta-analysis, by region in sub-Saharan Africa. Excluded studies were identified in a previous systematic review by Zurcher et al [2].

| **Included** | **Study / Region** | **Country** | **Setting** | **Age group** | **LTFU definition** | **Tracing method** | **Study period** | **No. traced**  **This study/ Zürcher 2017** |
| --- | --- | --- | --- | --- | --- | --- | --- | --- |
| **East Africa** |  |  |  |  |  |  |  |  |
| Yes | Geng 2015[3] | Kenya, Uganda, Tanzania | Urban | Adults | Missed appointment >90 days | Home visit | 2009-2012 | 579/991^*^ |
| Yes | Rachlis 2015[4] | Kenya | Urban | Adults and children | No visit >3 months | Home visit | 2009-2011 | 881/851 |
| Yes | Kiragga 2013[5] | Uganda | Urban | Adults | No visit for >3 months | Telephone, home visit | 2005-2007 | 163/406^**^ |
| No | Mekuria 2015[6] | Ethiopia | Urban | Adults | No visit for >1 month, | Telephone | 2009-2012 | 116 |
| No | Wubshet 2013[7] | Ethiopia | Urban | Adults | Missed appointment >3 months | Home visit | 2005-2010 | 551 |
| No | Makunde 2012[8] | Tanzania | Urban | Adults | No visit for >3 months | Home visit | 2006-2008 | 89 |
| No | Alamo 2012[9,10] | Uganda | Urban | Adults | No visit for >90 days | Home visit, buddy contact | 2001-2010 | 164 |
| No | Geng 2008[11] | Uganda | Rural | Adults | No visit for >6 months | Home visit | 2004-2007 | 128 |
| **West Africa** |  |  |  |  |  |  |  |  |
| Yes | Mben 2012[12] | Cameroon | Urban | Adults | Missed appointment >1 month | Telephone, home visit, relatives | 2006-2007 | 238/238 |
| No | Saka 2013[13] | Togo | Mostly urban | Adults | Not reported | Telephone | 2008-2011 | 1004 |
| No | Onoka 2012[14] | Nigeria | Mostly urban | Adults | Missed ≥3 appointments | Telephone, home visit | 2007-2007 | 150 |
| **Southern Africa** |  |  |  |  |  |  |  |  |
| Yes | Ardura-Garcia 2015[15] | Malawi | Urban | Children | Missed appointment ≥3 weeks | Home visit | 2006-2010 | 201/201 |
| Yes | Tweya 2013[1] | Malawi | Urban | Adults | Missed appointment >3 weeks | Telephone, home visit | 2006-2010 | 4558/4467 |
| Yes | Caluwaerts 2009[16] | Mozambique | Urban | Adults | No visit for >60 days | Home visit | 2002-2007 | 594/594 |
| No | Weigel 2011[17] | Malawi | Urban | Adults and children | Missed appointment >2 weeks | Telephone, home visit | 2002-2005 | 659 |
| No | Henriques 2012[18] | Malawi | Rural | Adults | Missed appointment >1 month | Home visit | 2004-2007 | 305 |
| No | Peltzer 2011[19] | South Africa | Mostly urban | Adults | Missed appointment ≥2 months | Telephone, home visit | 2007-2008 | 169 |
| No | McGuire 2010[20] | Malawi | Rural | Adults | Missed appointment >1 month | Home visit | 2004-2007 | 624 |
| No | McGuire 2010[20] | Malawi | Rural | Children | Missed appointment >1 month | Home visit | 2004-2007 | 32 |
| No | Bisson 2008[21] | Botswana | Urban | Adults | Missed appointment >1 month | Telephone, home visit | 2003-2003 | 68 |
| No | Krebs 2008[22] | Zambia | Urban | Adults | Missed appointment | Home visit | 2005-2005 | 654 |
| No | Dalal 2008[23] | South Africa | Urban | Adults | Missed appointment for ≥6 weeks | Telephone, home visit | 2004-2005 | 267 |
| No | Maskew 2007[24] | South Africa | Urban | Adults | Missed appointment | Telephone | 2006-2007 | 154 |
| No | Yu 2007[25] | Malawi | Rural | Mostly adults | No visit for >3 months | Home visit | 2004-2006 | 253 |
|  |  |  |  |  |  |  |  |  |

Included studies are shaded in grey.

* 579 patients with tracing outcomes were communicated.

**243 patients cART non-naïve at program enrolment were excluded from our analysis.

**References**

1. Tweya H, Feldacker C, Estill J, et al. Are they really lost? ‘True’ status and reasons for treatment discontinuation among HIV infected patients on antiretroviral therapy considered lost to follow up in Urban Malawi. PLoS One **2013**; 8:e75761.

2. Zurcher K, Mooser A, Anderegg N, et al. Outcomes of HIV-positive patients lost to follow-up in African treatment programmes. Trop Med Int Health **2017**; 22:375–387.

3. Geng EH, Odeny TA, Lyamuya RE, et al. Estimation of mortality among HIV-infected people on antiretroviral treatment in east Africa: a sampling based approach in an observational, multisite, cohort study. Lancet HIV **2015**; 2:e107–e116.

4. Rachlis B, Ochieng D, Geng E, et al. Implementation and operational research: evaluating outcomes of patients lost to follow-up in a large comprehensive care treatment program in western Kenya. J Acquir Immune Defic Syndr. **2015**; 68:e46-55.

5. Kiragga AN, Castelnuovo B, Musomba R, et al. Comparison of methods for correction of mortality estimates for loss to follow-up after ART initiation: a case of the Infectious Diseases Institute, Uganda. PLoS One **2013**; 8:e83524.

6. Mekuria LA, Prins JM, Yalew AW, Sprangers MAG, Nieuwkerk PT. Retention in HIV Care and Predictors of Attrition from Care among HIV-Infected Adults Receiving Combination Anti-Retroviral Therapy in Addis Ababa. PLoS One **2015**; 10:e0130649.

7. Wubshet M, Berhane Y, Worku A, Kebede Y. Death and seeking alternative therapy largely accounted for lost to follow-up of patients on ART in northwest Ethiopia: a community tracking survey. PLoS One **2013**; 8:e59197.

8. Makunde WH, Francis F, Mmbando BP, et al. Lost to follow up and clinical outcomes of HIV adult patients on antiretroviral therapy in care and treatment centres in Tanga city, North-Eastern Tanzania. Tanzan J Health Res **2012**; 14.

9. Alamo ST, Colebunders R, Ouma J, et al. Return to normal life after AIDS as a reason for lost to follow-up in a community-based antiretroviral treatment program. J Acquir Immune Defic Syndr **2012**; 60:e36-45.

10. Talisuna-Alamo S, Colebunders R, Ouma J, et al. Socioeconomic support reduces nonretention in a comprehensive, community-based antiretroviral therapy program in Uganda. J Acquir Immune Defic Syndr. **2012**; 59:e52-9.

11. Geng EH, Emenyonu N, Bwana MB, Glidden D V, Martin JN. Sampling-based approach to determining outcomes of patients lost to follow-up in antiretroviral therapy scale-up programs in Africa. JAMA **2008**; 300:506–7.

12. Mben JM, Kouanfack C, Essomba CN, et al. Operational research and HIV policy and guidelines: lessons from a study of patients lost to follow-up from a public antiretroviral treatment program in Cameroon. J Public Heal Policy **2012**; 33:462–477.

13. Saka B, Landoh DE, Patassi A, et al. Loss of HIV-infected patients on potent antiretroviral therapy programs in Togo: risk factors and the fate of these patients. Pan Afr Med J **2013**; 15:35.

14. Onoka CA, Uzochukwu BS, Onwujekwe OE, et al. Retention and loss to follow-up in antiretroviral treatment programmes in southeast Nigeria. Pathog Glob Health **2012**; 106:46–54.

15. Ardura-Garcia C, Feldacker C, Tweya H, et al. Implementation and Operational Research: Early Tracing of Children Lost to Follow-Up From Antiretroviral Treatment: True Outcomes and Future Risks. J Acquir Immune Defic Syndr **2015**; 70:e160-7.

16. Caluwaerts C, Maendaenda R, Maldonado F, Biot M, Ford N, Chu K. Risk factors and true outcomes for lost to follow-up individuals in an antiretroviral treatment programme in Tete, Mozambique. Int Health **2009**; 1:97–101.

17. Weigel R, Hochgesang M, Brinkhof MW, et al. Outcomes and associated risk factors of patients traced after being lost to follow-up from antiretroviral treatment in Lilongwe, Malawi. BMC Infect Dis **2011**; 11:31.

18. Henriques J, Pujades-Rodriguez M, McGuire M, et al. Comparison of methods to correct survival estimates and survival regression analysis on a large HIV African cohort. PLoS One **2012**; 7:e31706.

19. Peltzer K, Ramlagan S, Khan MS, Gaede B. The social and clinical characteristics of patients on antiretroviral therapy who are ‘lost to follow-up’ in KwaZulu-Natal, South Africa: a prospective study. SAHARA J **2011**; 8:179–186.

20. McGuire M, Munyenyembe T, Szumilin E, et al. Vital status of pre-ART and ART patients defaulting from care in rural Malawi. Trop Med Int Health **2010**; 15 Suppl 1:55–62.

21. Bisson GP, Gaolathe T, Gross R, et al. Overestimates of survival after HAART: Implications for global scale-up efforts. PLoS One **2008**; 3:1–6.

22. Krebs DW, Chi BH, Mulenga Y, et al. Community-based follow-up for late patients enrolled in a district-wide programme for antiretroviral therapy in Lusaka, Zambia. AIDS Care **2008**; 20:311–317.

23. Dalal RP, MacPhail C, Mqhayi M, et al. Characteristics and outcomes of adult patients lost to follow-up at an antiretroviral treatment clinic in Johannesburg, South Africa. JAIDS J Acquir Immune Defic Syndr **2008**; 47:101–107.

24. Maskew M, MacPhail P, Menezes C, Rubel D. Lost to follow up: contributing factors and challenges in South African patients on antiretroviral therapy. S Afr Med J **2007**; 97:853–857.

25. Yu JK-L, Chen SC-C, Wang K-Y, et al. True outcomes for patients on antiretroviral therapy who are &quot;lost to follow-up&quot; in Malawi. Bull World Health Organ **2007**; 85:550–4.
